# Supplementary material for: Triboelectric micromotors actuated by ultralow frequency mechanical stimuli
Source: Nat Commun. 2019 May 24;10:2309. doi: 10.1038/s41467-019-10298-7 (PMC6534612; doi:10.1038/s41467-019-10298-7)
Supplement: Supplementary file 1 — Supplementary Information [file 41467_2019_10298_MOESM1_ESM.pdf]

Supplementary Information

Triboelectric micromotors actuated by ultralow frequency mechanical stimuli

Hang *et al*.

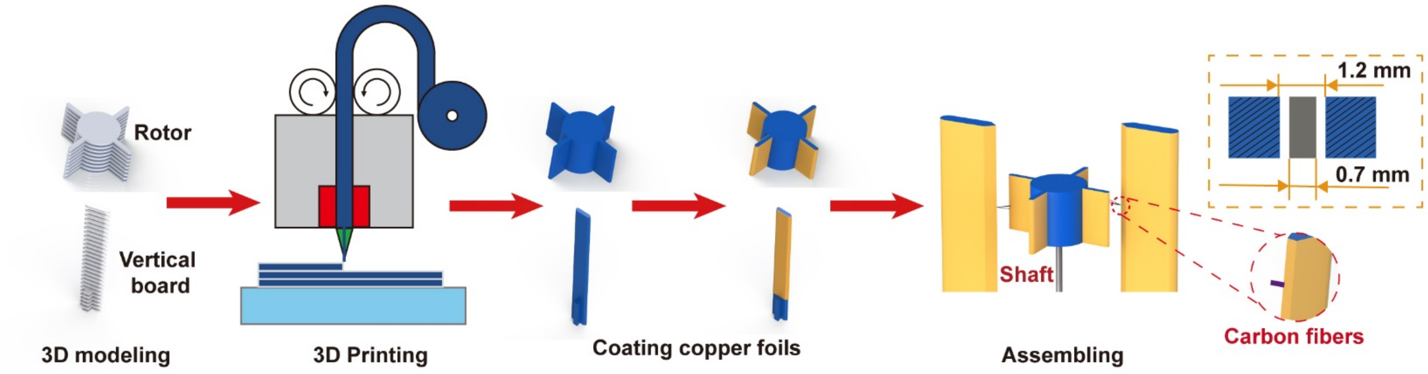

Supplementary Figure 1: Fabrication process of the micromotor.

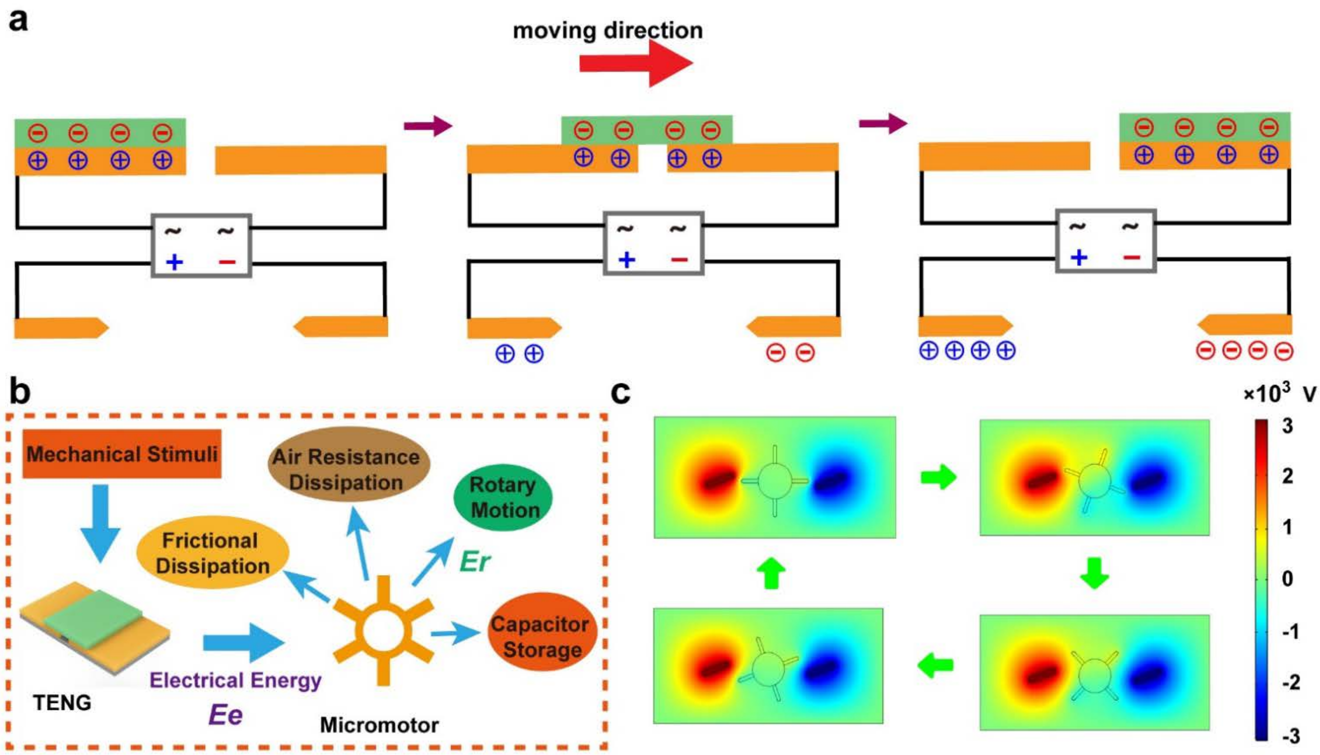

Supplementary Figure 2: Charges accumulation, energy transfer and voltage distribution simulation of the TENG. a Charges accumulating illustration of the TENG. **b** The energy conversion of the TM actuated by mechanical stimuli. **c** Voltage simulation when micromotor rotates.

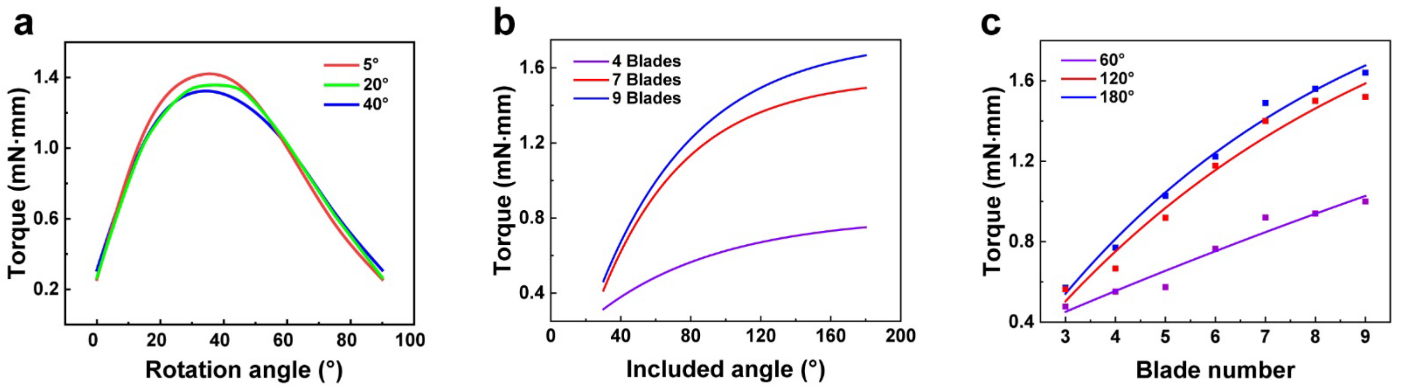

Supplementary Figure 3: Simulated electrostatic torque at different parameters. a Simulated relationship between electrostatic torque and rotation angle during a quarter circle at different tilt angles, with 4 blades and included angle of 180 degrees. **b** Simulated average electrostatic torque versus included angle at different blade number. **c** Simulated average electrostatic torque versus blade number at different included angle.

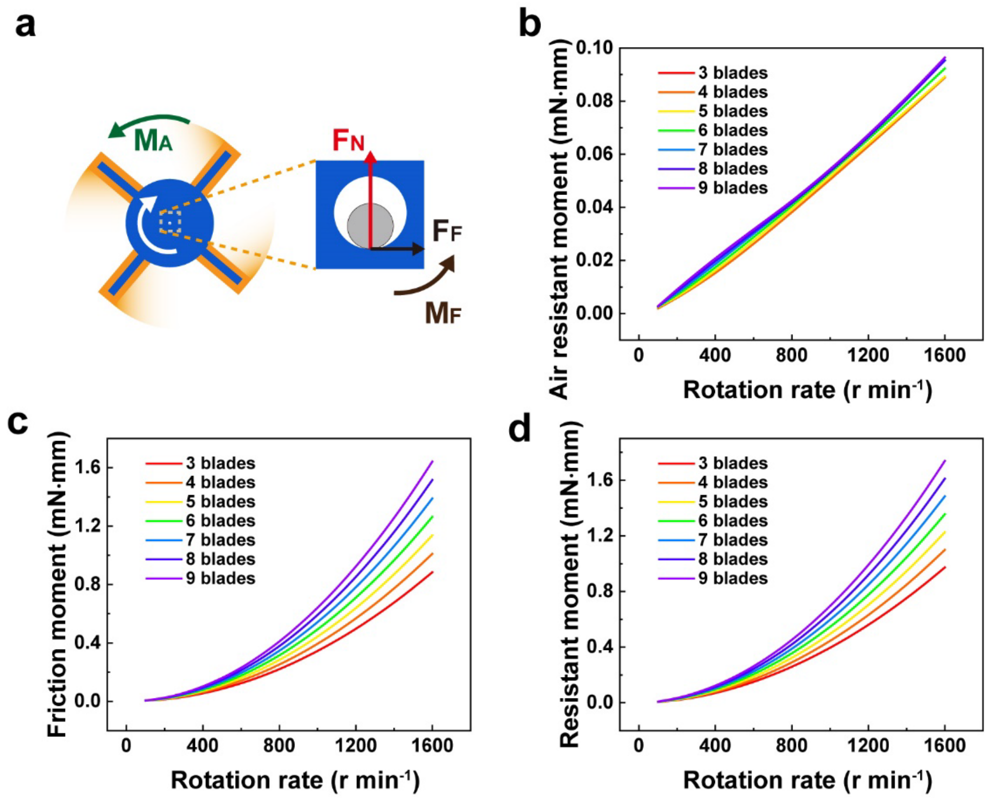

Supplementary Figure 4: Simulated resistant moment with different rotation rate. a Illustration of the resistant moments when the micromotor rotates. **b** Air resistant moment versus rotation rate at different blade number. **c** Friction moment versus rotation rate at different blade number. **d** Total resistant moment versus rotation rate at different blade number.

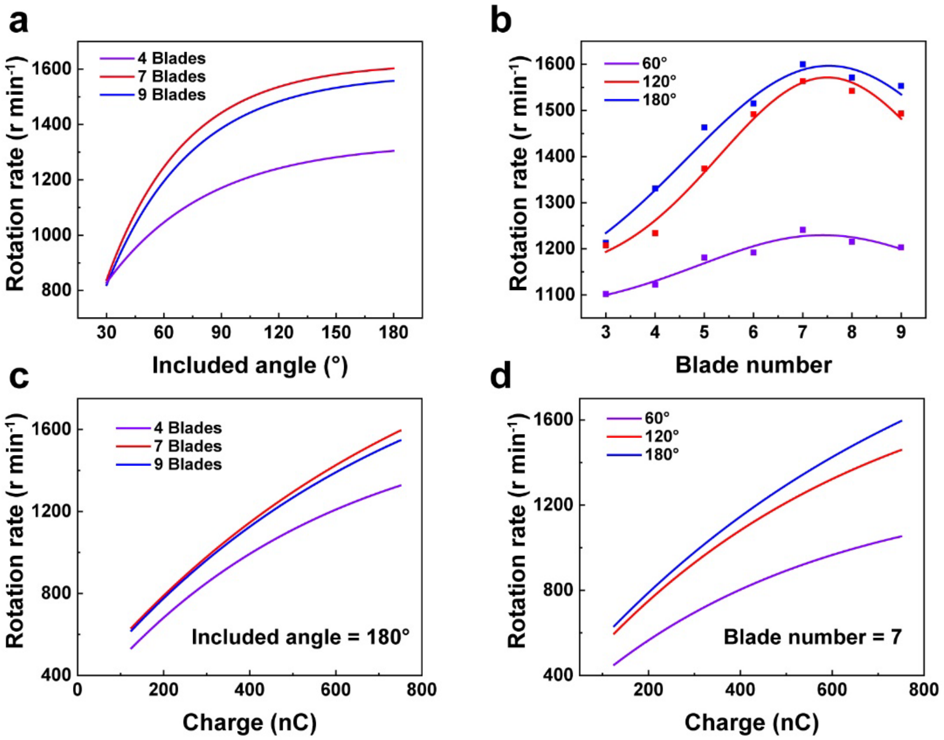

Supplementary Figure 5: Simulated rotation rate with different parameters. a Simulated rotation rate versus included angle at different blade number. **b** Simulated rotation rate versus blade number at different included angle. **c** Simulated rotation rate versus charge at different blade number. **d** Simulated rotation rate versus charge at different included angle.

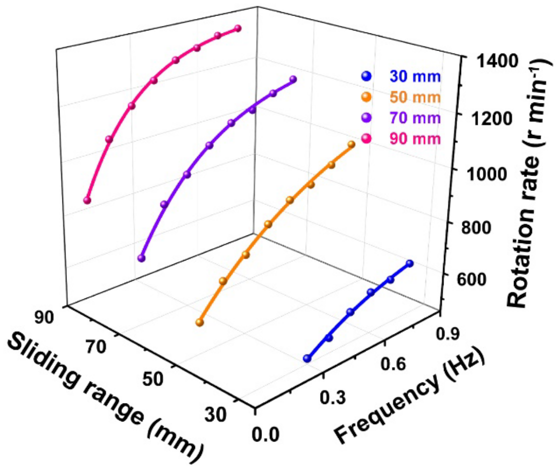

Supplementary Figure 6: Measured rotation rates with different sliding frequencies of the TENG at four different sliding ranges.

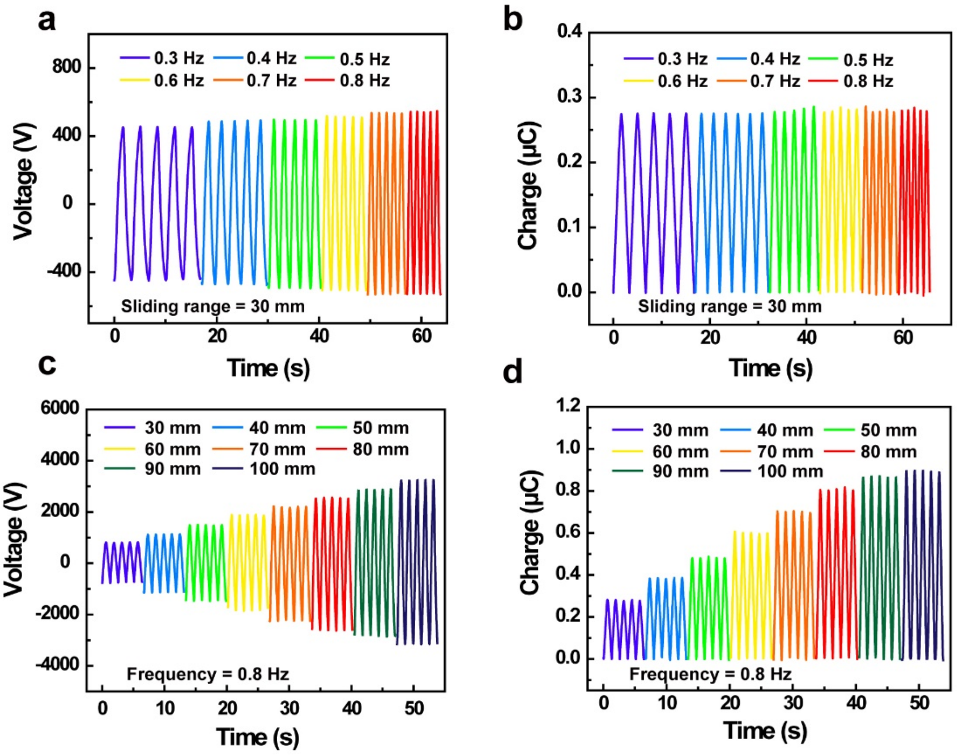

Supplementary Figure 7: Measured voltage and charge waveforms of the TENG with micromotor at different frequencies and sliding ranges. a, b Voltage and charge waveforms of the TENG with different frequencies at the sliding range of 30 mm, respectively. **c, d** Voltage and charge waveforms of the TENG with different sliding ranges at 0.8 Hz, respectively.

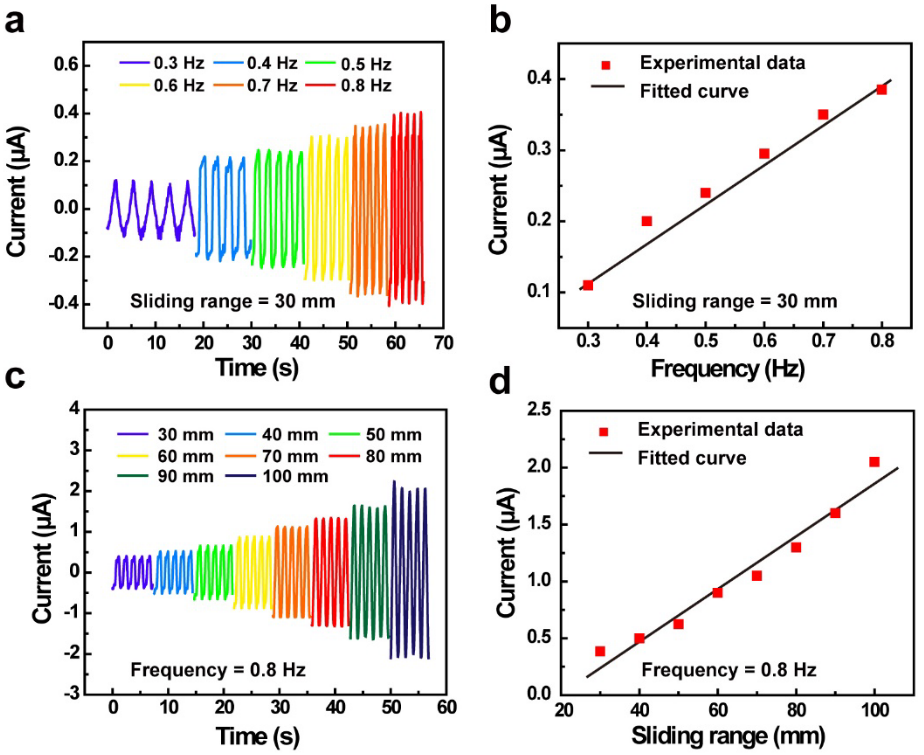

Supplementary Figure 8: Measured output current of the TENG with micromotor at different

frequencies and sliding ranges. a, b Current waveforms and peak values with different frequencies at the sliding range of 30 mm. **c, d** Current waveforms and peak values with different sliding ranges at 0.8 Hz.

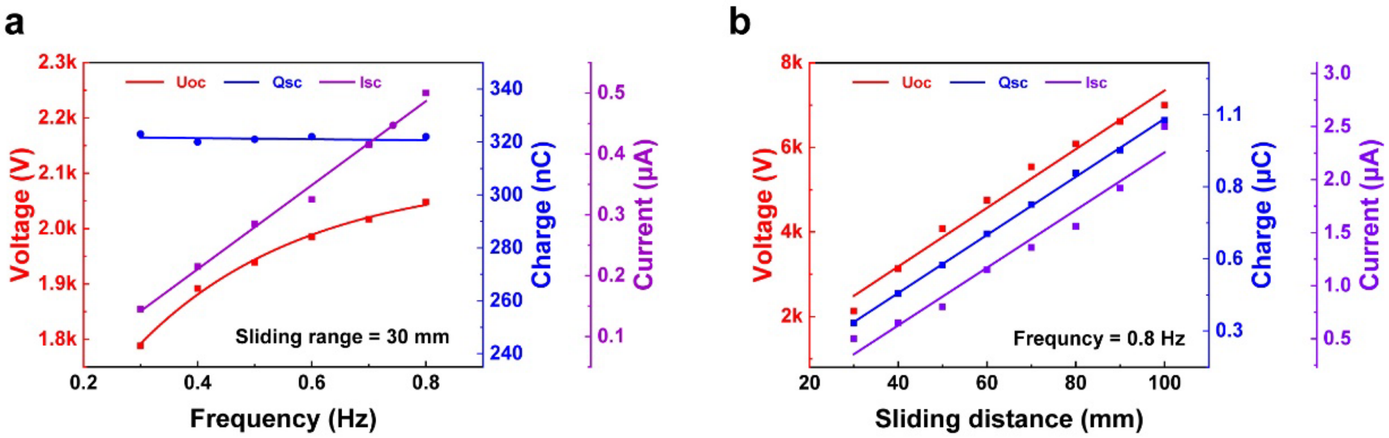

Supplementary Figure 9: Measured output characteristics of the TENG without micromotor. a Measured peak-to-peak open-circuit voltages, short-circuit transferred charges and short-circuit current of TENG with different frequencies at the sliding range of 30 mm. **b** Measured peak-to-peak open-circuit voltages, short-circuit transferred charges and short-circuit current of TENG with different sliding ranges at 0.8 Hz.

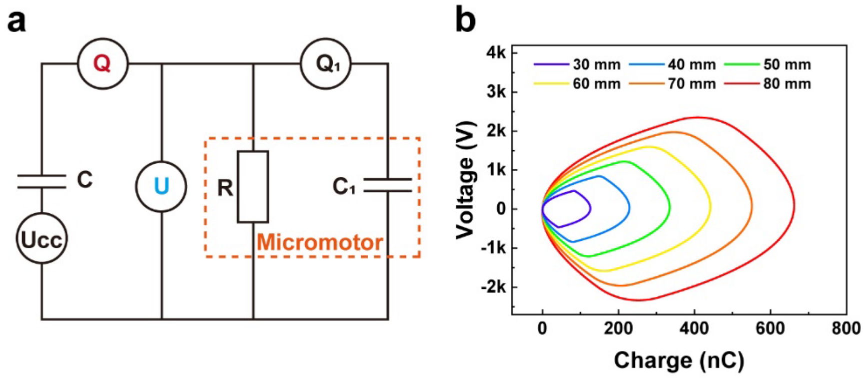

Supplementary Figure 10: ***U-Q*** **curves simulation of the TM. a** Equivalent circuit model of the TM. **b** Simulated *U-Q* curves with different sliding ranges at 0.8 Hz.

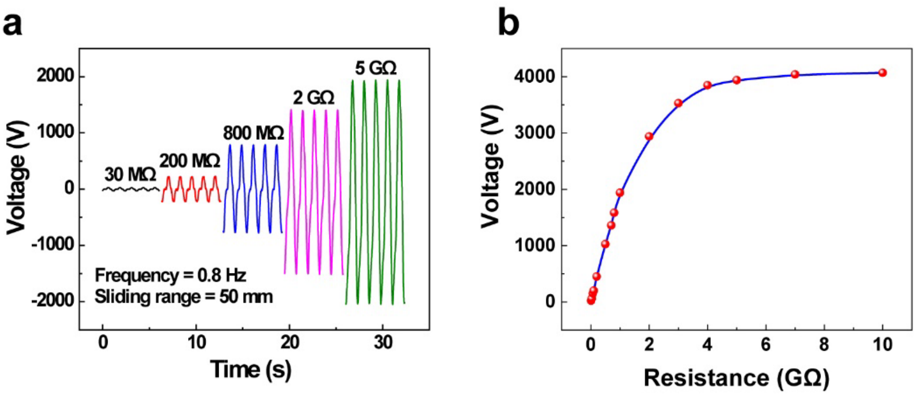

Supplementary Figure 11: Measured output voltage of the TENG on different load resistances.

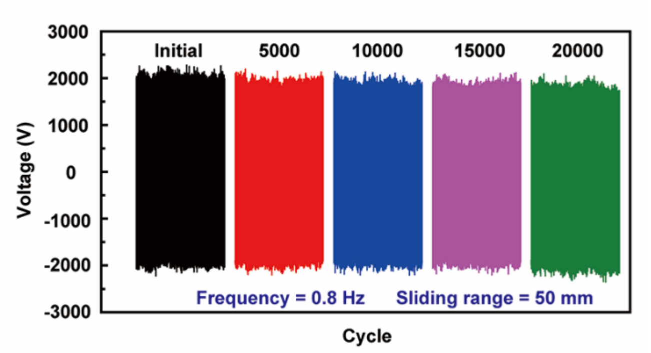

Supplementary Figure 12: The stability of sliding mode TENG.

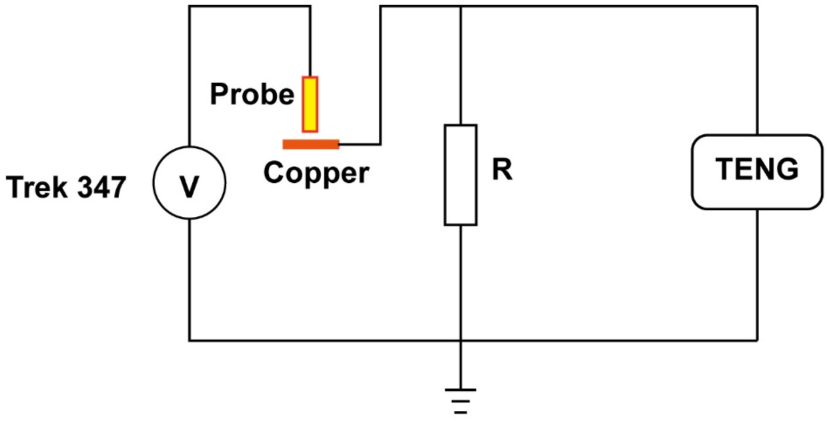

Supplementary Figure 13: Illustration of the load resistance measurement.

Supplementary Table 1: Summary of several low power consumption rotary micromotors.

Driving type	Material	Diameter (mm)	Speed (r min^{-1})	Power density (W m^{-3})	Driving frequency (Hz)	Efficiency (%)	Ref
Ultrasonic	PZT	60	60	2420	3.9 K	—	1
Ultrasonic	PZT	1.6	430	~267000	221 K	11	2
Optical	SU-8	0.01	160	~0.3	—	~10^{-17}	3
Optical	Liquid crystalline	0.008	660	~1.28	—	~10^{-18}	4
Microstreaming	SU-8	0.065	625	~11.9	15 M	~10^{-17}	5
Triboelectric	PLA	20	1300	~16	~1	41	This work

Supplementary Note 1: Rotating characteristics simulations of the TM.

When the micromotor is rotating, the driving moment comes from the electrostatic torque provided by the TENG, while the resistant moment mainly consists of the air resistant moment and the friction moment as illustrated in Supplementary Figure 4a. When the driving and resistant moments are equal, the micromotor can rotate at the balanced state, which can be described as:

$$M_E = M_A + M_F \quad (1)$$

where M_E is the electrostatic torque, M_A is the air resistant moment and M_F is the friction moment.

We first set the accumulated charge quantity on the vertical electrodes as 750 nC. The electrostatic torques of the TM with different parameters are simulated and calculated by COMSOL software, such as tilt angle, include angle and blade number, which are shown in Supplementary Figure 3. The simulation results have shown that the tilt angle has nearly no effects on the electrostatic torque, while the electrostatic torque increases with larger include angle and blade number.

Meanwhile, the air resistant moments are simulated and calculated by ANSYS software, as shown in Supplementary Figure 4b, in which the air resistant moment grows up with larger rotation rate and the distinction between different blade numbers is not obvious. As for the friction moment, theoretical calculations are performed as following:

$$F_N = m\omega^2(R - r) \quad (2)$$

$$F_F = \mu \cdot F_N \quad (3)$$

$$M_F = F_F \cdot R \quad (4)$$

where m is the mass of the rotor, ω is the angular velocity of the rotor, μ is the friction factor between the shaft and rotor, F_N is the rotational centripetal force of the shaft, and F_F is the friction force on the shaft. The symbols R and r are the radius of the hole in the rotor and the shaft. The results are shown in Supplementary Figure 4c, which reveals that larger blade number and rotation rate result in greater friction moment. By combine air resistant moment and friction moment, the total resistant moment can be obtained in Supplementary Figure 4d, in which the friction moment is dominant and the air resistant moment can be ignored.

For the balanced state that the driving and resistant moments are equal, the rotation rate can be calculated with different included angle, blade number and charge, respectively. As depicted in Supplementary Figure 5a, the larger included angle can give rise to the larger rotation rate. Although more blade can generate larger driving moment, the corresponding resistant moment is

larger for the greater mass and friction force. Therefore, the optimal blade number is calculated to be 7 as shown in Supplementary Figure 5b.

Besides the structural parameters, the effects of the accumulated charges on the rotation rate have also been taken into account. In Supplementary Figure 5c and 5d, we simulated the rotation rate versus charge quantity from 125 nC to 750 nC at different blade number and included angle, the results have verified that the micromotor rotates faster with more charges and the variation trend tends to be saturated.

Supplementary Note 2: Equivalent circuit model of the TM and *U*-*Q* curves simulation.

From the working mechanism of the TM, we can find out that two electric characteristics in the rotating process. One is that the charges can be accumulated on the vertical electrodes, while the other is that the current can be generated by charges transferring from one electrode to the other. Therefore, we assume that the micromotor can be approximately equivalent to a small capacitance and a large resistance in parallel, and the equivalent circuit model of the TM is depicted in Supplementary Figure 10a. Accordingly, we can derive the following equations:

$$\frac{dQ(t)}{dt} = \frac{dQ_1(t)}{dt} + \frac{U(t)}{R} \quad (5)$$

$$U(t) = \frac{Q_1(t)}{C_1} \quad (6)$$

$$U_{CC} - \frac{Q(t)}{C} = \frac{Q_1(t)}{C_1} \quad (7)$$

where Q is the trunk charges and Q_1 is the charges pass through the capacitor C_1. The symbols C and C_1 are the capacitance of the TENG and micromotor. U_{CC} is the built-in potential of the TENG, and U is the applied voltage on the micromotor. Here, R, C and C_1 are set to 600 MΩ, 0.6 nF and 0.01 nF, respectively. For the convenience of circuit modeling and simulation, we have ignored the rectifier and changed the negative voltage to the positive, which will not affect the accuracy of the applied voltage on the micromotor. Given the initial condition of $Q_1 = 0$ (t=0), we can solve equations (1-3) as:

$$Q(t) = e^{-\frac{t}{R(C+C_1)}}\left(U_{CC}(0) \cdot C + \int_0^t \frac{Ce^{-\frac{z}{R(C+C_1)}}\left(\frac{dU_{CC}(z)}{dz}C_1 \cdot R + U_{CC}(z)\right)}{R(C+C_1)}dz\right) \quad (8)$$

$$U(t) = U_{CC}(t) - e^{-\frac{t}{R(C+C_1)}}\left(U_{CC}(0) + \int_0^t \frac{e^{-\frac{z}{R(C+C_1)}}\left(\frac{dU_{CC}(z)}{dz}C_1 \cdot R + U_{CC}(z)\right)}{R(C+C_1)}dz\right) \quad (9)$$

where z is a temporary variable of integration. Here, for the sliding motion of the TENG, we define a periodic mechanical motion equation:

$$x(t) = \frac{x_m}{2}(1 - cos2\pi ft) \quad (10)$$

where x_m is the sliding range and f is the mechanical frequency of the TENG. Utilizing the minimum achievable charge reference state (MACRS) [6], the relevant U_{CC} with sliding displacement $x(t)$ can be calculated[7]. With the numerical calculation by Python, the *U*-*Q* curves with different sliding ranges at the frequency of 0.8 Hz is displayed in Supplementary Figure 10b, in which the output electric energy from the TENG in one cycle, described as the encircled area of *U*-*Q* curve, increases at larger sliding range.

Supplementary Note 3: Comparison with several low power consumption rotary micromotors

In Supplementary Table 1, we have summarized several low power consumption rotary micromotors, such as ultrasonic, optical and microstreaming types. In the comparison, we can find that the TM has both higher efficiency and rotate speed. It has also higher power density than the optical and microstreaming micromotors. Although the ultrasonic micromotors powered by AC power source have higher power density than the TM, the speeds are low and the driving frequencies are usually up to KHz or MHZ. While the TM can be actuated by the mechanical stimuli with an ultralow frequency as low as 0.1 Hz, instead of the external high voltage power supply. Therefore, the TM has provided a novel method for a high-speed scanner actuated in ultralow frequency, which are much different and superior to previous micromotors.

Supplementary References

1. An D, Yang M, Zhuang X, Yang T, Meng F, Dong Z. Dual traveling wave rotary ultrasonic motor with single active vibrator. *Appl. Phys. Lett.*, **110**, 143507 (2017)
2. Cagaty S, Koc B, Moses P, Uchino K. A piezoelectric micromotor with a stator of φ= 1.6 mm and l= 4 mm using bulk PZT. *Jpn. J. Appl. Phys.* **43** 1429–1433 (2004)
3. Kelemen L, Valkai S, Ormos P. Integrated optical motor, *Appl. Opt.* **45**, 2777–2780 (2006)
4. Ito K, Frusawa H, Kimura M. Precise switching control of liquid crystalline microgears driven by circularly polarized light. *Opt. Express* **20**, 4254−4259 (2012)
5. Kao J, Wang X, Warren J, Xu J Attinger D. A bubble-powered micro-rotor: conception, manufacturing, assembly and characterization, *J. Micromech. Microeng.* **17**, 2454–2460 (2007)
6. Niu, S. *et al.* Theory of freestanding triboelectriclayer-based nanogenerators. *Nano Energy*. **12**, 760−774 (2015).
7. Zi, Y. *et al.* Standards and figure-of-merits for quantifying the performance of triboelectric nanogenerators. *Nat. Commun.* **6,** (2015).
