# Supplementary material for: Triboelectric micromotors actuated by ultralow frequency mechanical stimuli
Source: Nat Commun. 2019 May 24;10:2309. doi: 10.1038/s41467-019-10298-7 (PMC6534612; doi:10.1038/s41467-019-10298-7)
Supplement: Supplementary file 3 — Description of Additional Supplementary Files [file 41467_2019_10298_MOESM3_ESM.docx]

### Description of Additional Supplementary Files

**File Name: Supplementary Movie 1**

**Description: Micromotor actuated by TENG in ultralow frequency**

**File Name: Supplementary Movie 2**

**Description: Barcode recognition by hand motion**

**File Name: Supplementary Movie 3**

**Description: Moving obstacle detection by tire rolling**
